# Lessons learned from hybrid surgery with preoperative coil embolization for an aberrant artery in pulmonary sequestration

**DOI:** 10.1186/s40792-021-01277-6

**Published:** 2021-08-24

**Authors:** Keita Nakanishi, Masaki Goto, Shota Nakamura, Toyofumi Fengshi Chen-Yoshikawa

**Affiliations:** Department of Thoracic Surgery, University Graduate School of Medicine, 65 Tsurumai-cho, Showa-ku, Nagoya, 466-8550 Japan

**Keywords:** Pulmonary sequestration, Aberrant artery, Coil embolization, Hybrid surgery

## Abstract

**Background:**

The optimal management of an aberrant artery in pulmonary sequestration (PS) is controversial. Several studies have shown that hybrid surgery with preoperative coil embolization for an aberrant artery and surgical resection of the sequestrated lung is effective. However, there are no clear indications for the procedure.

**Case presentation:**

A 68-year-old woman without any complaints was diagnosed with right intralobar PS, which was supplied by an aberrant artery from the thoracic aorta, via computed tomography performed during a medical examination. In addition, lung adenocarcinoma was detected over the border between the right upper and middle lobes. Preoperative coil embolization was performed by an interventional radiologist the day before surgery to decrease the risk of severe intraoperative hemorrhage. On the following day, bi-lobectomy of the right upper and middle lobes for lung adenocarcinoma with systemic lymph node dissection and segmentectomy of the sequestrated lung with thoracotomy was performed. Although no active hemorrhage was observed during surgery, the aberrant artery was challenging to dissect using an energy device due to the presence of an intravascular coil. Eventually, the coil stump was exposed, and it was cut with scissors. The postoperative course was uneventful.

**Conclusions:**

We reported the pitfall of the hybrid surgery for intralobar PS. Preoperative coil embolization can prevent fatal intraoperative hemorrhage. If embolization is performed using a coil for an aberrant artery supplied from the thoracic aorta, where and how to dissect the aberrant artery should be cautiously determined based on preoperative images, with consideration of the presence of an intravascular coil.

## Background

Pulmonary sequestration (PS) is a rare respiratory malformation characterized by a mass in the lung parenchyma with an aberrant systemic arterial supply. Surgical resection is generally recommended for the disease regardless of the presence of symptoms [1]. Thoracic surgeons must consider how to treat a fragile aberrant artery due to the risk of serious and potentially fatal hemorrhage during surgery [2]. However, the optimal management of an aberrant artery is controversial. Several studies have shown that hybrid surgery with preoperative coil embolization for an aberrant artery can effectively reduce the risk of serious intraoperative hemorrhage [3–7]. However, there are no clear indications for the procedure. Herein, we present a case of hybrid surgery with preoperative coil embolization for an aberrant artery and surgical resection of the sequestrated lung. Moreover, the indications and challenges of the procedure were discussed.

## Case presentation

**Fig. 1:**
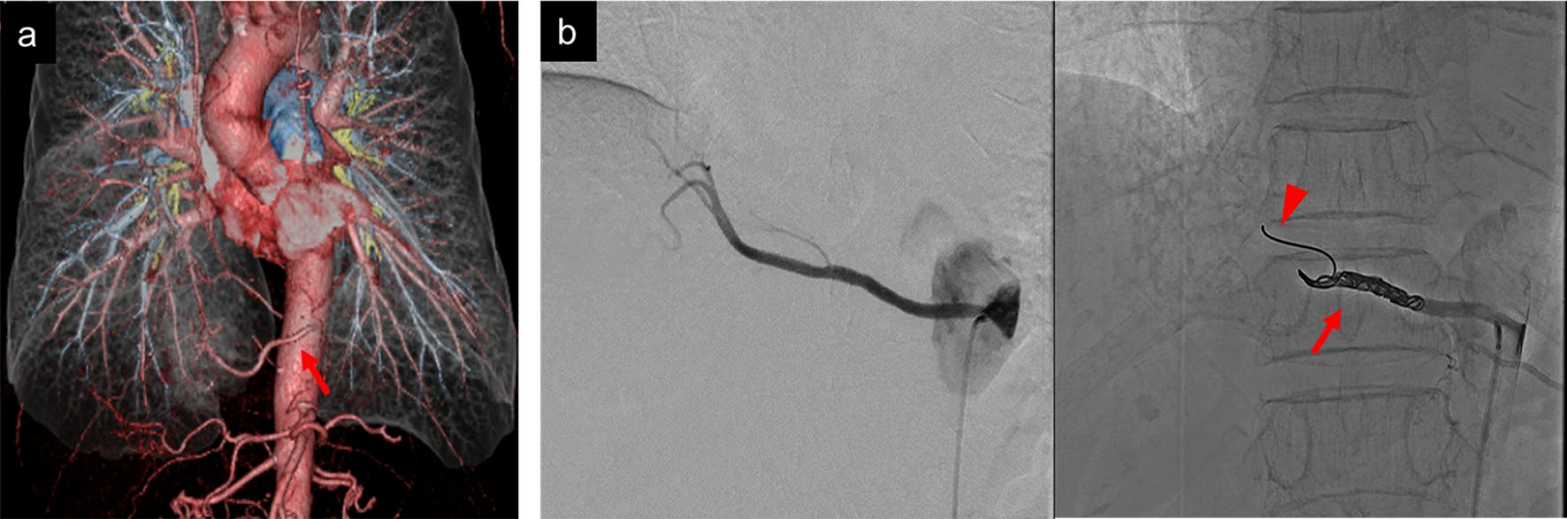
**a** Three-dimensional reconstructed images showing the aberrant artery from the thoracic aorta (red arrow). **b** Angiography and embolization of the aberrant artery using a coil. The arrow shows coil embolization of the aberrant artery. The arrowhead indicates the coil stump using the first branch of the aberrant artery as an anchor

A 68-year-old woman without any complaints was diagnosed with right intralobar PS via contrast-enhanced computed tomography scan performed during a medical examination. Repeated infections were not observed. The aberrant artery originated from the thoracic descending aorta and measured approximately 4 mm in diameter (Fig. 1a). In addition, lung adenocarcinoma was detected over the border between the right upper and middle lobes (S3/S4) and diagnosed based on bronchoscopy. Hence, simultaneous fractional lung resection and radical resection of lung tumor via thoracotomy were planned. Coil embolization was conducted by an interventional radiologist a day before surgery to decrease the risk of serious intraoperative hemorrhage. A 4-Fr sheath was placed in the right femoral artery. POD coil (Penumbra Incorporated, Alameda, CA, USA) measuring 4 mm × 30 cm was placed using the first branch of the aberrant artery as an anchor (Fig. 1b). Angiography after embolization showed complete occlusion. The patient did not experience any symptoms after coil embolization.

**Fig. 2:**
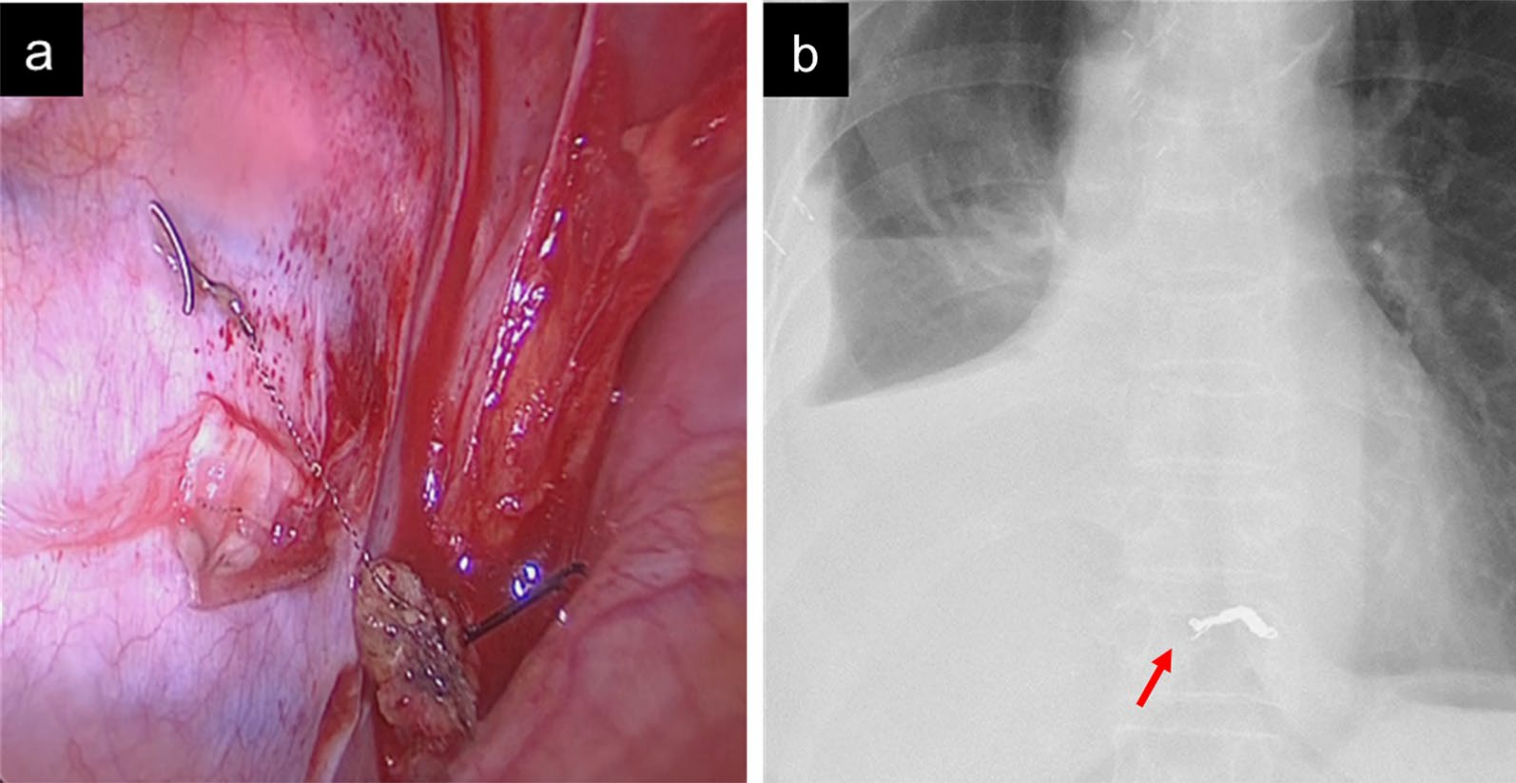
**a** Intraoperative finding. After the arterial wall was dissected, the coil stump was exposed. **b** Postoperative chest radiography showing that the coil stump was cut (arrow) and that a part of the coil remained

On the following day, bi-lobectomy of the right upper and middle lobes for lung adenocarcinoma with systemic lymph node dissection and segmentectomy of the sequestrated lung with thoracotomy was performed. The aberrant artery was found and ligated when the pulmonary ligament was detached. However, dissection using an energy device could not be completely performed due to an error. Although the arterial wall was finally dissected, it was cut at the location, where the coil was found, and the coil stump used as an anchor happened to expose (Fig. 2a). No hemorrhage was observed, and the exposed coil was cut with scissors. After dissecting the vein according to the sequestrated lung, 5 mg of indocyanine green was administered intravenously to detect the boundary between the normal and sequestrated lung. The postoperative course was uneventful. Postoperative radiography revealed that the remaining aberrant artery was coil embolized (Fig. 2b).

## Discussion

This case report showed the challenges experienced when performing hybrid surgery with preoperative coil embolization for an aberrant artery originating from the thoracic aorta in PS. A previous study has reported two cases of massive intraoperative hemorrhage due to aberrant arterial injury [8]. Thus, in patients who undergo surgery for PS, the appropriate and safe management of an aberrant artery is the most important factor considered by thoracic surgeons. With the development of endovascular techniques, several reports have shown that preoperative coil embolization is effective in decreasing the risk of serious intraoperative hemorrhage [3–7]. However, challenges associated with the procedure, similar to those observed in the current case, have not been discussed in previous studies.

To date, there is no established treatment guideline for PS, and data about hybrid surgery is limited. In previous cases, patients at high risk of recurrence and hemorrhage, such as those with large pulmonary lesions and inflammatory changes due to repeated infections, commonly undergo hybrid surgery [4, 5]. Although hybrid surgery with preoperative coil embolization was performed for an aberrant artery of 3.5 mm [4], the precise cutoff diameter of the aberrant artery for hybrid surgery remains unclear. The method of embolization and not the indication may change depending on the aberrant artery thickness. Previous studies have reported that coils were thought to be more suitable in small, tortuous, and branched vessels, whereas plugs were particularly suited for large, short, and high-flow vessels [4, 9]. In addition, hybrid surgical procedures with stent graft rather than with coil embolization was commonly performed for PS with the aneurysmal anomalous artery [10, 11]. Therefore, for patients with a larger vessel diameter or aneurysm formation, hybrid surgery with plugs and stent graft may be a good indication. Furthermore, the preoperative coil embolization may be useful for patients with PS who undergo video-assisted thoracoscopic surgery to prevent the risk of bleeding in poor visual point, such as under the diaphragm [3, 5]. From January 2009 to December 2020, 15 patients underwent surgical resection for PS at our institution. Then, three (20%) had preoperative coil embolization for an aberrant artery. The indication for the procedure was based on the surgeon’s discretion. In the current case, we performed preoperative coil embolization to decrease the risk of serious intraoperative hemorrhage, because radical resection of lung cancer was simultaneously performed.

In several cases, hybrid surgery with preoperative coil embolization is feasible and effective in preventing intraoperative active hemorrhage. However, in patients with an aberrant artery originating from the thoracic aorta, as in the current case, where and how to dissect the aberrant artery should be assessed, with consideration of the presence of an intravascular coil. That is because the length of the aberrant artery originating from the thoracic aorta was not long enough compared with the one originating from the abdominal aorta. Savic et al. showed that 73.9% of aberrant arteries were supplied from the thoracic aorta in 373 patients with PS, whereas 18.7% originated from the abdominal aorta [12]. In the current case, the point of dissection in the aberrant artery was not discussed, and the intravascular coil in the location to be dissected was not assumed. If embolization is performed using coil for the aberrant artery supplied from the thoracic aorta, the aberrant artery must be dissected after confirming the location of the intravascular coil via palpation or radiography, and the vessel should be sufficiently exfoliated to the periphery. Otherwise, Sakai et al. proposed that endostapling with intravascular coils for an aberrant artery was a simple and safe technique [5]. Thus, studies with a larger number of patients should be conducted to determine the safety and efficacy of hybrid surgery and its indications.

## Conclusions

Although preoperative coil embolization for an aberrant artery in PS can prevent intraoperative active hemorrhage, thoracic surgeons have to carefully consider and discuss indications of hybrid surgery with interventional radiologists. If embolization is performed using a coil for an aberrant artery supplied from the thoracic aorta, where and how to dissect the aberrant artery should be cautiously determined based on preoperative images, with consideration of the presence of an intravascular coil.

## Data Availability

Data supporting the findings of this study are available from the corresponding author upon reasonable request.

## Abbreviation

PS: Pulmonary sequestration
